# Supplementary material for: CRISPR/Cas9-Mediated Multiple Knockouts in Abscisic Acid Receptor Genes Reduced the Sensitivity to ABA during Soybean Seed Germination
Source: Int J Mol Sci. 2022 Dec 18;23(24):16173. doi: 10.3390/ijms232416173 (PMC9784318; doi:10.3390/ijms232416173)
Supplement: Supplementary file 1 [file ijms-23-16173-s001.zip › Supplementary Material Figure S1-S6_20221111183450.pdf]

Supplementary Material

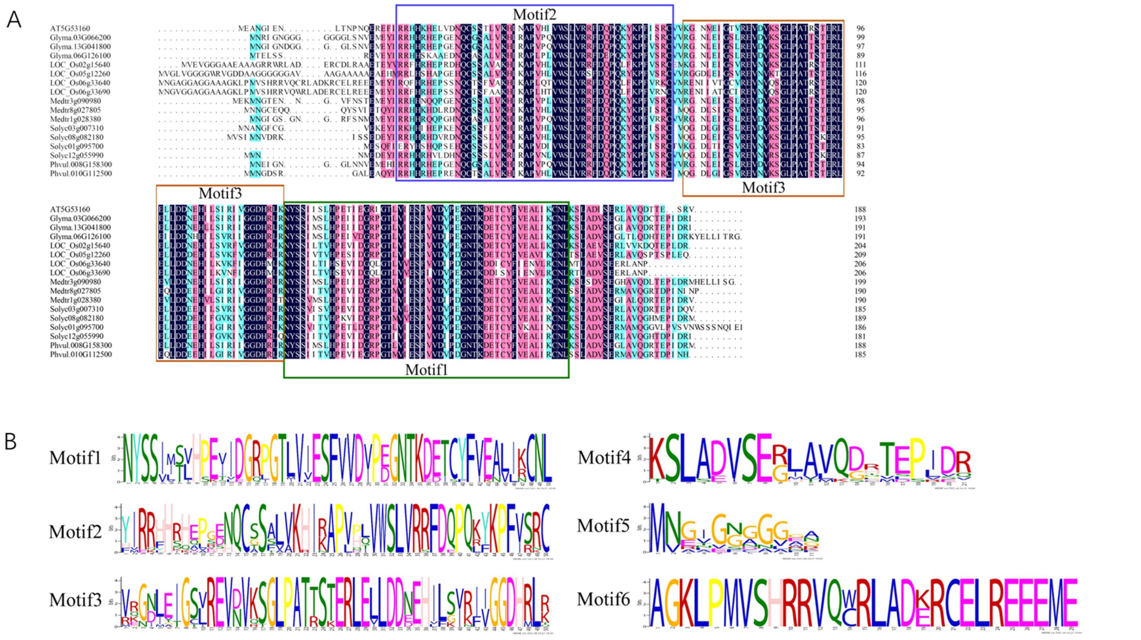

Supplementary Figure 1. The gene conserved motifs of PYLs. **(A)**Three predicted motifs are represented by different colored boxes, **(B)**Motif LOGO showing below. All motifs were identified using the MEME website(http://meme-suite.org/) with the complete amino acid sequences of PYLs.

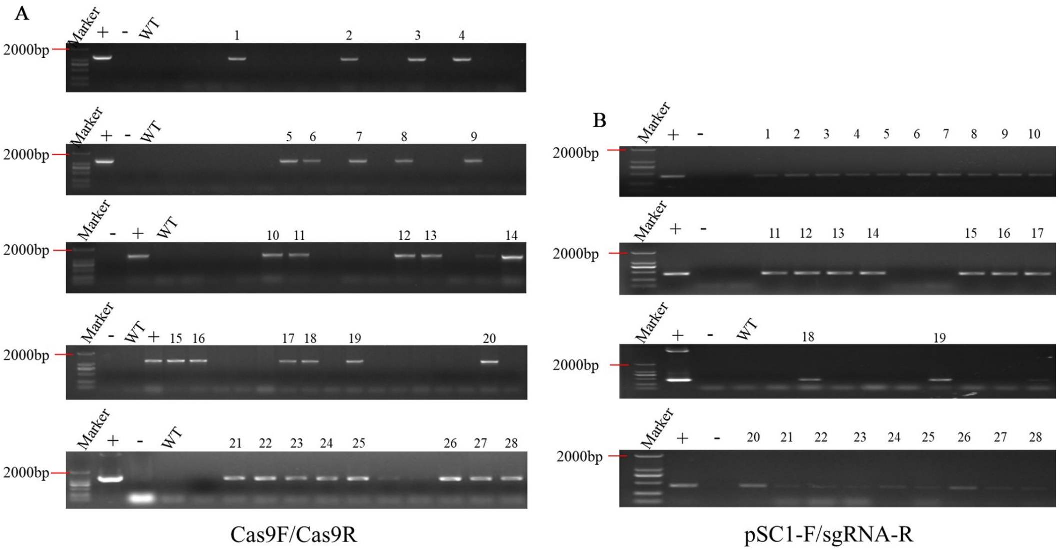

Supplementary Figure 2. Homozygous of soybean generated by the CRISPR/Cas9 system in the T_1 generation. **(A)** PCR-based genotype of the Cas9 gene and gRNA of the transgenic soybean plants. "+" indicates positive control. "-" indicates negative control. Marker indicates a DNA ladder. **(B)** Sequences and peaks at target sites of WT and *gmpyl17/19-1*, *gmpyl17/18/19-1*, and *gmpyl17/18/19-2* mutant. Dashes indicate deleted nucleotides. Nucleotides in red indicate PAM. Red arrowheads indicate mutation locations.

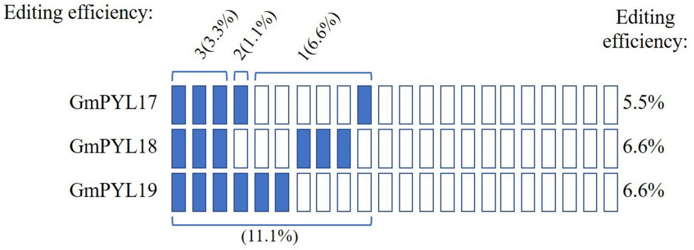

Supplementary Figure 3. Quantification of editing events measured by sequencing from soybean transformants. Each column represents one transgenic event; blue rectangles indicate target genes with editing; white rectangles indicate wild-type.

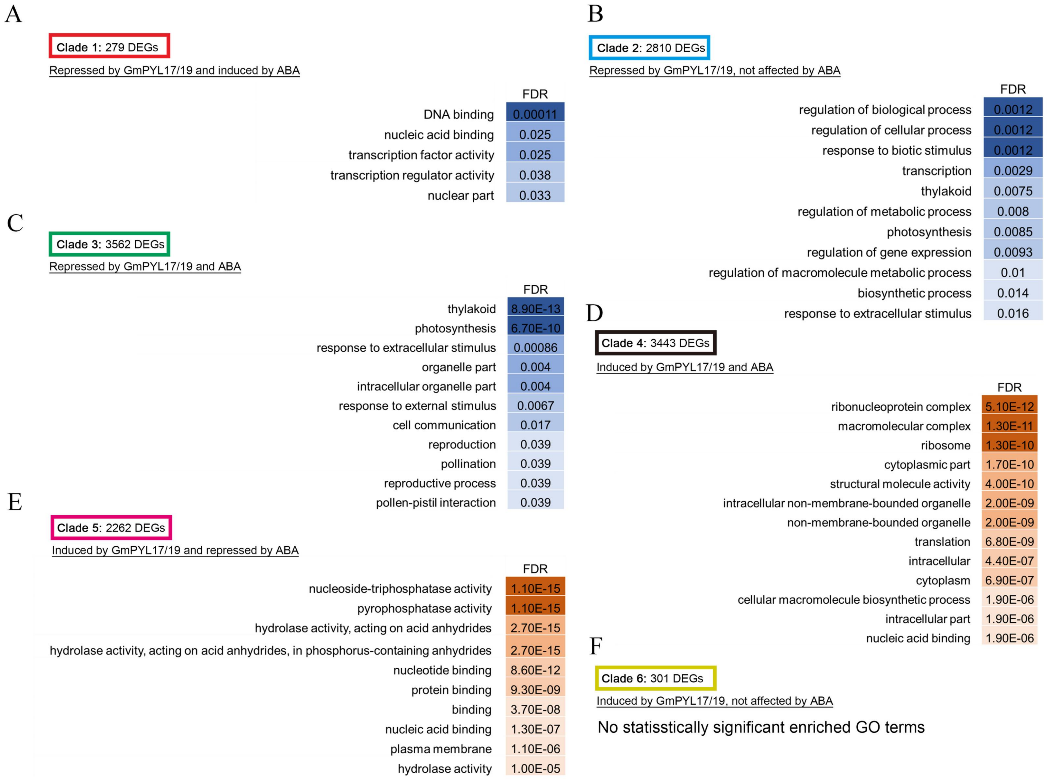

Supplementary Figure 4. Transcriptome analysis of the *gmpyl17/19-1* mutant and WT under normal(CK) and ABA stress(10μM ABA treatment). **(A–C).** Significantly enriched GO terms of the Clades 1–3 DEGs repressed by *GmPYL17/19* identified in Figure 7C. DEGs in Clades 1–3 were upregulated DEGs in the *gmpyl17/19-1* mutant and they could be further grouped into Clades 1–3 based on their response to ABA stress. **(D–F)** Significantly enriched GO terms of the Clades 4–6 DEGs induced by GmPYL17/19 identified in Figure 7D. DEGs in Clades 4–6 were down regulated DEGs in the *gmpyl17/19-1* mutant and they could be further grouped into Clades 4–6 based on their response to ABA stress. Values indicate the FDR values for the corresponding GO terms. All the GO enrichment analysis results of the six clades DEGs identified in Figures 7C and D were list in Supplemental Table S4.

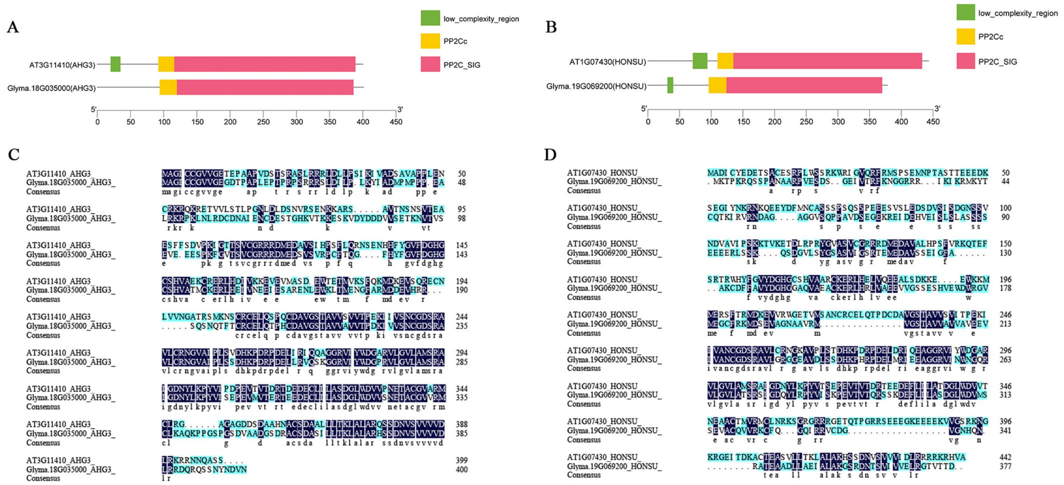

Supplementary Figure 5. Comparison of *AHG3* and *HONSU* gene from *Arabidopsis thaliana* and *Glycine max*. **(A, B)**. Conserved domains of *AHG3* and *HONSU* from *Arabidopsis thaliana* and *Glycine max*. All AHG3 and HONSU gene contains the PP2C domain. **(C, D)** Alignment of the amino acid sequences of *AHG3 from* and *HONSU*. The dark blue font background represents the conserved amino acid.

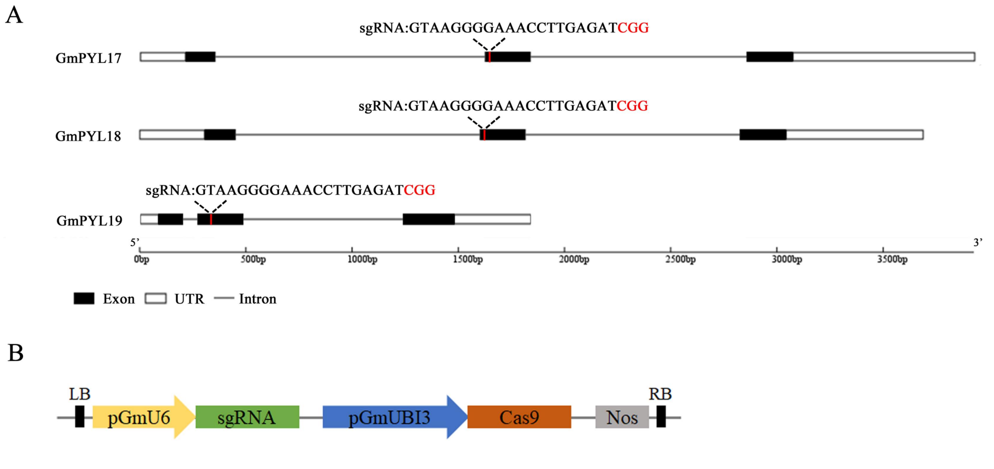

Supplementary Figure 6 . GmPYLs gene structure and target sequence locations. Schematic diagram of simultaneous site-directed mutagenesis of GmPYL17, GmPYL18, and GmPYL19 using the CRISPR/Cas9 technology. **(A)** Exon–intron structure of GmPYL17, GmPYL18, and GmPYL19. Black boxes indicate exons and red stripes are editing sites. Continuous lines indicate introns and white boxes indicate untranslated regions (UTRs). Nucleotide sequences indicate regions targeted by the sgRNAs designed in this study; nucleotides in red indicate the proto-spacer adjacent motif (PAM). Gene structure analyzed online by the GENE Structure display (GSDS 2.0) website (http://gsds.gao-lab.org/)(Hu et al., 2015) **(B)** Schematic of the T-DNA region of the targeting vector designed for mutagenesis of the PYLs genes using the CRISPR/Cas9 system. GmUbi3 indicates the Glycine max Ubi3 promoter. GmU6 indicates the Glycine max U6 promoter. Cas9 gene was expressed by 35S promoter. LB, left border; RB, right border.

Supplementary Table 1. Number of the conserved motif amino acid of PYLs protein.

Supplementary Table 2 . Primers used to identify transgene positive.

Supplementary Table 3. Primers for GmPYL17、GmPYL18、GmPYL19 genes containing amplified fragments of the gRNA sequence region.

Supplementary Table 4 .The GO term involved in the article

Supplementary Table 5 . Primers used for qRT-PCR in this study
